# The impact of songs with prosocial lyrics on implicit cognition and prosocial behavior: a prospective event-related brain potential study

**DOI:** 10.3389/fpsyg.2025.1426891

**Published:** 2025-06-10

**Authors:** Qiujian Xu, Siqi Liu, Meihui Li, Xiaoyu Wang, Junrui Li, Xinran Yuan, Miaomiao Yang, Mingyi Yang, Zhenxu Jiang, Qiaoqiao Gou, Ningning Liu, Jiaqi Han, Dan Yang, Xiubo Ren

**Affiliations:** ^1^Art Healing and Cognitive Science Research Center, Department of Music, School of Arts and Design, Yanshan University, Qinhuangdao, China; ^2^YSU & DCU Joint Research Centre for the Arts, College of Music and Performing Arts, Daegu Catholic University, Daegu, Republic of Korea; ^3^School of Mechanical Engineering, Tianjin University of Commerce, Tianjin, China

**Keywords:** music with prosocial lyrics, prosociality, implicit cognition, event-related potential, the general learning model

## Abstract

**Background:**

Music with prosocial lyrics is a significant area of study in music psychology. Based on the General Learning Model, such music can effectively enhance prosocial behaviors. However, little is known about the neural correlates of the impact on prosocial behavior of short-term exposure to music with prosocial lyrics. Previous research has primarily used self-report measures to explore the relationship between music and prosocial cognition and behavior. However, these measures can be influenced by social desirability biases when dealing with sensitive issues such as moral behavior. The study of implicit cognition can effectively avoid these biases and has thus attracted widespread attention.

**Objective:**

This study is the first to investigate the electrophysiological characteristics of the relationship between exposure to music with prosocial lyrics and enhanced altruistic behavior and to elucidate the effects of such music on implicit prosocial cognition.

**Methods:**

This laboratory study will recruit 45 college students, who will be tasked with listening to either music with prosocial lyrics or neutral music. We will then use the Single Category Implicit Association Test (SC-IAT) paradigm combined with event-related potentials (ERP) to investigate the impact of music with prosocial lyrics on participants' implicit prosocial cognition and further reveal the predictive power of implicit cognition on prosocial behavior (measured by assessing the level of voluntary unpaid participation in subsequent experiments).

**Results:**

The findings of this study will provide neuroscientific evidence on how music with prosocial lyrics influences prosocial behavior through cognitive processes and clarify the effectiveness of music with prosocial lyrics in enhancing implicit prosocial cognition and behavior. In addition, these findings will not only deepen the understanding of the relationship between music and social behavior but also provide theoretical foundations and practical guidance for education, psychological interventions, and strategies to improve social behavior, thereby promoting the application of music in fostering social harmony.

## 1 Introduction

Music with prosocial lyrics has received extensive attention from researchers as an artistic genre that combines aesthetic values and social functions. Prosocial lyrics are mainly characterized by the promotion of peace, love, sharing, and positive social interaction (Hong et al., 2023). Music with prosocial lyrics demonstrates music's potential as a powerful social tool for fostering and encouraging cooperative interactions and ethical behavior among individuals and groups (Greitemeyer, 2009; Hong et al., 2023; Ma et al., 2023; McDonald et al., 2022; Ruth, 2017, 2019; Ruth and Schramm, 2021; Yu et al., 2019). Previous research has revealed that music with prosocial lyrics facilitates extrinsic prosocial cognition (a measure of participants' self-reports), but it is not known whether such music can affect implicit cognitive processes. Compared with explicit cognition, implicit cognition is mainly processed using the episodic processing system, suggesting that implicit prosocial cognition is more susceptible to external situational factors (Tao and Li, 2002). However, few studies have explored the relationship between music with prosocial lyrics and implicit cognition. Only one study, by Ding et al. (2015), focuses on this relationship in a group of Chinese middle school students. Studies comparing participants' performances on the Lexical Decision Task (LDT) (Yu et al., 2019) and the Word Categorization Task (WCT) (Ruth, 2019) found that those hearing prosocial songs had more prosocial implicit cognition than those exposed to neutral songs. However, the neural mechanisms of this phenomenon have not been investigated. The proposed study aims to explore the impact of short-term exposure to prosocial music on prosocial behavior and further reveal its effects on implicit prosocial cognition using event-related potentials (ERPs).

An ancient Chinese proverb states, “To change customs and transform manners, nothing is better than music,” suggesting the intuition that music education may be an effective way of changing and cultivating moral qualities and maintaining social order. The General Learning Model (GLM) proposes that all situational factors from the external environment, such as music, can impact individual behavior (prosocial behavior) through a series of learning mechanisms (Buckley and Anderson, 2006). Prosocial behavior, a positive psychological trait, is a crucial part of a healthy personality and promotes the socialization of individuals (Huang et al., 2023). College students, in the early stages of adulthood, begin the transition from being a “natural person” in a biological sense to becoming a “social person” who takes on certain social responsibilities and adapts to social life during their university years (Wang et al., 2021). Acts of kindness not only benefit college students' academic performance (King et al., 2024), social interactions, and physical and mental health (Le et al., 2018; Miles et al., 2021; Peacock, 2022) but are also fundamental to achieving sustainable human development (Neaman et al., 2018). Music listening is a common activity for college students (Hu et al., 2014, 2021; Kennedy et al., 2008), with studies showing that they listen to music for more than 4 hours daily (Rubin et al., 2001). Therefore, it is particularly necessary to thoroughly explore how music affects college students' prosocial cognition and further verify its predictive power regarding prosocial behavior.

The dual attitudes model categorizes altruistic cognition into implicit prosocial attitudes and explicit prosocial attitudes (Aydinli et al., 2016; Guo et al., 2022; Resh et al., 2019; Wu et al., 2018; Zhang et al., 2019). When sensitive issues such as moral goodness are involved, implicit measures are effective in reducing participants' bias and measuring cognitive attitudes that are not easily expressed (Hahn and Gawronski, 2015). Numerous studies support the idea that implicit measures are superior to explicit measures as predictors of behavior (Greitemeyer, 2009; Hofmann et al., 2005; Huntjens et al., 2014; Nosek et al., 2011). Greitemeyer conducted the first study on the mediating effects of music with prosocial lyrics, finding that prosocial music induced more prosocial cognitions. For example, the group exposed to prosocial songs enumerated more prosocial thoughts. In experimental scenarios or real-life situations where good behavior is mostly performed under conditions of scarce resources or weak motivation, it is more useful and effective to explore the effects of prosocial music on implicit prosocial cognition as opposed to explicit prosocial cognition.

The Implicit Association Test (IAT) paradigm demonstrates unique advantages in measuring implicit cognition toward socially sensitive topics. The IAT relies on participants' response times rather than self-reports, reducing the impact of social desirability effects. Karpinski and Steinman improved the traditional IAT paradigm by designing the Single Category Implicit Association Test (SC-IAT) paradigm, which addresses the IAT's inability to evaluate a single object and simplifies the operational procedure, making it easier to understand (Karpinski and Steinman, 2006). SC-IAT (Single Category Implicit Association Test) is a commonly used tool to measure implicit cognition and indirectly reflect implicit attitudes. The D-value obtained from calculating participants' scores represents the participant's implicit effect; a higher D-value indicates a higher level of prosocial behavior in the participant's implicit cognition. Research with Chinese samples has shown that the SC-IAT demonstrates superior reliability and validity compared to other tools for measuring implicit cognition, such as the EAST (The Extrinsic Affect Simon Task), GNAT (GO/NO-GO), and ST-IAT (The Single-Target Implicit Association Test) (Wen and Zuo, 2007). Therefore, the SC-IAT paradigm provides a robust experimental framework for measuring individuals' implicit prosocial cognition following music interventions in this study.

The recording and analysis of event-related potentials (ERPs) represents a neuroscience technique with high temporal resolution that can be used to study the temporal dynamics of information processing in prosocial decision-making (Carlson et al., 2016; Xiao et al., 2015). No studies have yet investigated changes in implicit prosocial cognition and their neurocognitive processes after short-term exposure to music. Related studies have shown that ERP components of different cognitive processes exhibit high sensitivity in the IAT. For example, during the early stages of decision-making, the early ERP component is associated with selective attention and perceptual processes in the N1. The N1 is related to whether stimuli are congruent with self-judgments, with larger N1 amplitudes being elicited by stimuli that are incongruent with the self (Li et al., 2015). N2 is a widely observed ERP component that appears in the frontal region within a time window of 200–300 ms after stimulus presentation (Sun et al., 2021). When stimuli conflict with an individual's expectations, the amplitude of N2 increases significantly (Chen et al., 2025; Ji and Li, 2025; Portengen et al., 2022). Specifically, when individuals are faced with inconsistent or conflicting information, they require more cognitive effort to process these conflicts. For example, in the Stroop task, the N2 amplitude is larger in compatible conditions than in incompatible conditions (Wei et al., 2021; Wu et al., 2021). More importantly, N2 is also considered an emotional marker of prosocial behavior, as the N2 amplitude is significantly positively correlated with high empathy (Balconi and Canavesio, 2013). There are several psychological explanations for N2, one of which, particularly relevant to the Implicit Association Test (IAT), suggests that N2 reflects the degree of response conflict during task execution (Yeung et al., 2004). Studies related to the IAT have shown that compared to compatible trials, incompatible trials produce greater response conflict, whichis reflected in larger (more negative) N2 amplitudes (Williams and Themanson, 2011). For instance, Xiao et al. (2015) used the IAT to study implicit prosocial behavior and found that under incompatible conditions (self-related vocabulary vs. non-prosocial vocabulary), the N2 amplitude increased, indicating stronger conflict responses and suggesting that individuals rate themselves as more prosocial in their implicit self-assessments. N4 is defined as a negative peak within the 350–450 ms latency window (Chen et al., 2018). The amplitude of N4 can serve as a valuable neural indicator of implicit self-positivity bias (Tipura et al., 2025). When encountering semantically inconsistent words or sentences (e.g., “The boy ate the sofa”), the amplitude of N400 is significantly larger compared to when viewing semantically consistent sentences (e.g., “The boy ate the apple”) (Williams and Themanson, 2011). This phenomenon indicates that the amplitude of N400 is highly sensitive to semantic conflicts, such as syntactic or lexical inconsistencies. In the Implicit Association Test (IAT), incompatible trials (e.g., words that do not match their categories) can lead to cognitive conflict, thereby triggering larger N400 amplitudes. During the mid-stage of prosocial stimulus processing, the P200, which peaks at 200 ms in the frontal region of the brain, is closely associated with implicit cognition. The brain automatically matches the received visual information with internal representations, and the amplitude of the P200 is positively correlated with the degree of matching (Evans and Federmeier, 2007). The parietally distributed late positive component (LPC; sometimes considered a sustained P3 response and thus labeled P3-like or P3b-like response) is elicited at ~300 ms latency and continues to the end of the stimulus (Lou et al., 2021). Some studies have applied the ERP technique in IAT or IAT-like tasks, finding that congruent trials elicit significantly larger LPC amplitudes than incongruent trials (Coates and Campbell, 2010; Forbes et al., 2012; Williams and Themanson, 2011). We believe that conducting research on the relationship between music and implicit prosocial cognition and providing neurocorrelational evidence will open up a neglected pathway in the current field of study. This research will primarily focus on the N1, N2, N4, P200, and LPC components in ERPs.

Previous research has suggested that the formation of implicit attitudes is a slower process than that of explicit attitudes and that they are not easily changed because they are formed gradually through experience and extensive learning. Traditionally, it was believed that prosocial cognition could not be altered through short-term interventions. However, recent research has demonstrated that prosocial cognition can indeed be modified through external interventions. For example, Dasgupta and Greenwald found that repeatedly presenting participants with photos of disliked White individuals and respected Black individuals resulted in reduced implicit preference for White individuals through repetitive exposure to the images. In a second experiment, researchers presented photos of respected elderly individuals and disliked young individuals, which effectively reduced the participants' implicit preference for young people to a certain extent (Dasgupta and Greenwald, 2001).

Additionally, numerous studies have demonstrated the strong ability of implicit cognition to predict behavior. For example, a stronger implicit association between “self” and “death” vocabulary predicted suicidal behaviors (Nock et al., 2010; Nock and Banaji, 2007). A meta-analysis of the predictive validity of 184 tests using implicit association found positive predictive effects across all domains of evaluation (Greenwald et al., 2009). Therefore, the proposed study will further investigate whether prosocial cognition effectively predicts prosocial behavior, providing theoretical support for the predictive validity of implicit cognition on behavior. After participants perceived the study to be over, they were asked if they would be willing to assist with further research; how much time they committed was used as a measure of prosocial behavior. This measure was modified from that utilized by Greitemeyer (2009).

The proposed study will use the SC-IAT paradigm to analyze the impact of prosocial music on implicit prosocial cognition and, for the first time, employ ERP technology to observe its cognitive processes. Based on the GLM theoretical framework, the study will use music type as the independent variable, prosocial behavior (voluntary participation time in the experiment) as the dependent variable, and prosocial cognition (D-scores and brainwave amplitudes) as the mediating variables. Based on previous research findings, we propose the following hypotheses:

Hypothesis 1: We hypothesize that listening to prosocial songs enhances implicit prosocial cognition, meaning individuals exposed to prosocial music will associate themselves more with prosocial words compared to those exposed to neutral music. In the SC-IAT paradigm, participants in the prosocial song condition will show higher D-score values than those in the neutral song condition.
Hypothesis 2: We hypothesize that prosocial songs enhance implicit prosocial cognition and affect the N1, P200, and LPC. ERP results show that under compatible conditions, the prosocial music group exhibits larger P200 and LPC amplitudes and smaller N1 amplitudes than the neutral music group. Under incompatible conditions, the trend reverses.
Hypothesis 3: We hypothesize that listening to prosocial songs will boost prosocial behavior. Participants in the prosocial song condition will show more willingness to engage in further experiments and invest more time compared to those in the neutral song condition.

## 2 Methods

### 2.1 Pilot study

**Figure 1:**
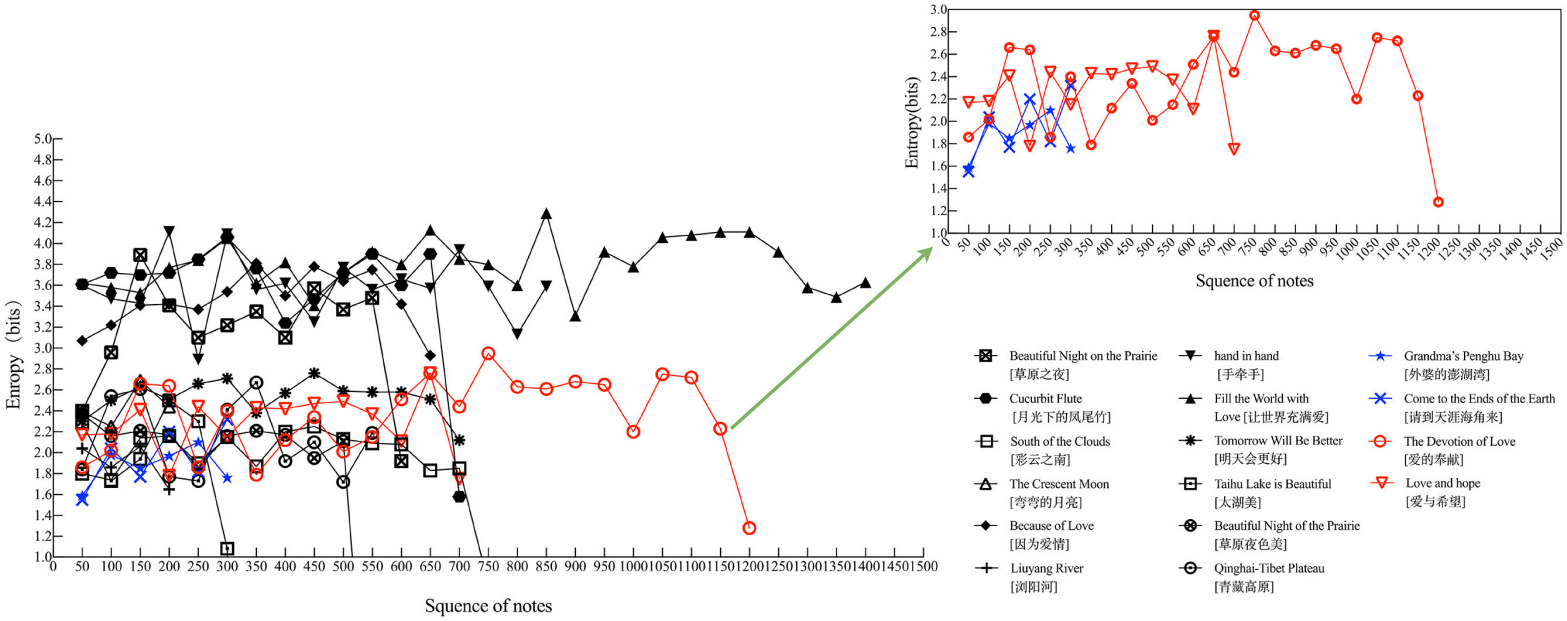
The sequential change of the Shannon entropies of songs.

It is crucial to consider various musical attributes that influence listeners' cognitive responses. The structural elements of music influence individuals' physical perception and emotional expression, with factors like sound intensity, pitch, tempo, and harmonic transitions contributing to the overall complexity of musical compositions (Gündüz, 2023). By reviewing previous studies on prosocial music materials (Hong et al., 2023; Yu et al., 2019), we selected 11 pieces of music, considering factors such as tonality and rhythm, and calculated the complexity of the music using Shannon entropy. Shannon entropy, a fundamental concept in information theory, serves to quantify uncertainty and randomness and holds relevance across diverse fields, including physics, psychology, and musicology. In the realm of music, Shannon entropy offers a means to measure the complexity of musical compositions and discern distinctions among musical genres (Li et al., 2024). Following the methodology outlined by Gündüz (2023), the Melody Shannon Entropy calculation was performed for each of the 11 selected pieces of music. The specific formula used for this calculation is provided in Appendix 1. Notably, this calculation encompassed all notes within the score, excluding ornaments lacking temporal significance; the resulting value was defined as the entropy of the piece. To further refine our analysis, we also determined separate entropy values for the entire piece (Melody Shannon Entropyoverall, SNO), the soprano voice (Melody Shannon Entropyright hand, SNR), and the bass voice (Melody Shannon Entropyleft hand, SNL). Figure 1 depicts the successive trends of entropy in the 11 pieces. The x-axis represents the frequency of occurrence of different notes, and the y-axis the corresponding entropy values. Based on the entropy trends of the 11 pieces shown in Figure 1, we selected two neutral and two prosocial music pieces with similar trends. The selected neutral music pieces are “Come to the Ends of the Earth” and “Grandma's Penghu Bay,” while the prosocial music pieces are “The Love of Devotion” and “Love and Hope.”

To ensure the effectiveness of the music materials used in the experiment, we plan to recruit 37 participants without a background in music to evaluate the selections. Based on previous research (Hong et al., 2023; Yu et al., 2019), participants used a self-report questionnaire to rate each piece of music on dimensions such as prosocial nature, feelings of love, and arousal. They listened to two pieces of music with prosocial lyrics and two with neutral lyrics and answered questions using a 7-point scale (0 = not related at all, 7 = highly related). All the musical pieces maintain consistent tonality, all are in major keys, and their time signatures are also consistent, with all pieces in 4/4 time. Paired-sample *t*-tests revealed a significant difference in the prosocial level between songs with prosocial lyrics (*M* = 6.24, SD = 0.56) and songs with neutral lyrics (*M* = 1.83, SD = 0.85), *t*_(37)_ = 21.05, *p* < 0.001. Conversely, there was no significant difference in arousal [*t*_(37)_ = −0.87, *p* = 0.39] and love levels [*t*_(37)_ = −0.44, *p* = 0.66). To ensure that the selected prosocial lyrics are more prosocial than the neutral lyrics, we ultimately chose the music based on the prosocial scores. As a result, we selected two prosocial songs (“The Devotion of Love” and “Love and Hope”) and two neutral songs (“Come to the Ends of the Earth” and “Grandma's Penghu Bay”) as the final musical materials. All data from the pilot study are available at the Open Science Federation (DOI 10.17605/OSF.IO/UWMV5).

These selections underwent editing to ensure uniformity in length, and all songs featured Chinese lyrics. The lyrics of the chosen songs were not presented in the textual materials of the SC-IAT paradigm, thus avoiding potential interference with the experimental conditions.

### 2.2 Participants

An independent statistician conducted an a priori power analysis to determine the necessary sample size. This study adopts a randomized crossover design. Based on previous research (Long et al., 2023), G^*^Power 3 was used to calculate the required sample size. With an expected average effect size of 0.20 and a predicted power of 0.8, it is estimated that a sample of 45 participants will be needed. The participant pool will consist of 45 right-handed students from Yanshan University, China. Participation in the study will be voluntary, with all participants providing informed consent. Eligibility criteria for formal experimentation include normal or corrected-to-normal vision, absence of recent medication usage, and no history of neurological defects or damage, such as language impairment. Furthermore, participants will not have been informed of the study's objectives, and none will have a background in a related field, such as psychology or musicology. Individuals with hearing impairments were specifically excluded from participation. Compensation for participation was provided either in the form of course credit or monetary reimbursement for their time. The study was approved by the Institutional Review Board of Qinhuangdao First People's Hospital.

### 2.3 Materials

#### 2.3.1 Music

*Neutral songs*. “Come to the Ends of the Earth” is a classic song that portrays the natural beauty and cultural richness of Hainan Province. The lyrics, “Come to the Ends of the Earth, where flowers and fruits abound,” highlight the region's lush landscapes. “Grandma's Penghu Bay” is a classic campus song that expresses admiration for the beautiful and beloved homeland of Grandma's Penghu Bay. The lyrics, “The evening breeze gently brushes Penghu Bay, white waves chase the beach, there are no coconut trees decorating the setting sun, only an expanse of blue sea,” evoke a serene and picturesque coastal scene.

*Prosocial songs*. “Devotion of Love” emerges as a quintessential piece characterized by themes of love, often regarded as emblematic of Chinese public welfare songs. The lyrics “As long as everyone gives a little love, the world will become a beautiful Earth” express a call and yearning for love. “Love and Hope” is a motivational relief song specially composed for the Wenchuan earthquake in China. The song emphasizes the power of love and hope in helping people overcome difficulties and rebuild their homes. The lyrics, “When love and hope cast the blazing sun, yesterday's tears will evaporate with time,” express a strong belief in the future.

#### 2.3.2 Word

There was a total of 20 two-character Chinese words in the prosocial cognition SC-IAT task. The target categories consisted of ten items, five for “self” [e.g., “我”(me), “我的”(mine)“我们”(we)“自己”(oneself)“咱们”(we or us)] (Zhang et al., 2022). The attribute categories also consisted of ten items, five for “prosocial” [e.g.,“关爱”(solicition)、“奉献”(dedication)、“支持”(support)、“帮助”(hand)、“保护”(safeguard)] and five for “non-prosocial” [(e.g.,“攻击”(target)“辱骂”(vituperation)“拒绝”(rejection)“欺骗”(deceptive)“藐视”(look down on)] (He and Zhu, 2016).

### 2.3.3 Scales

#### 2.3.3.1 Demographic information

Demographic statistics serve as crucial control variables. These variables include age, gender, nationality, profession, education, and socioeconomic status (SES), and music preferences. Additionally, prosocial tendency measures (PTM), as defined by Carlo and Randall (Carlo et al., 2003), are incorporated into the analysis.

#### 2.3.3.2 Music material

Participants will be tasked with evaluating the music on four dimensions: familiarity, prosociality (defined within the study), liking, and arousal. Each dimension is rated using a 5-point Likert-type scale, ranging from 1 (not at all) to 5 (very much). To ensure consistency in familiarity ratings, participants will be instructed to listen to both pieces of music on the day before the experiment.

#### 2.3.3.3 The modified differential emotions scale (mDES)

Emotions were assessed using the Modified Differential Emotions Scale (mDES), originally developed by Izard (2013) and later adapted by Cohn et al. (2009). Comprising 20 items, the mDES identifies 10 positive and 10 negative emotions. Responses are recorded on a five-point Likert scale, ranging from 1 (“Not at all”) to 5 (“Extremely”).

### 2.4 Experimental design and procedure

**Figure 2:**
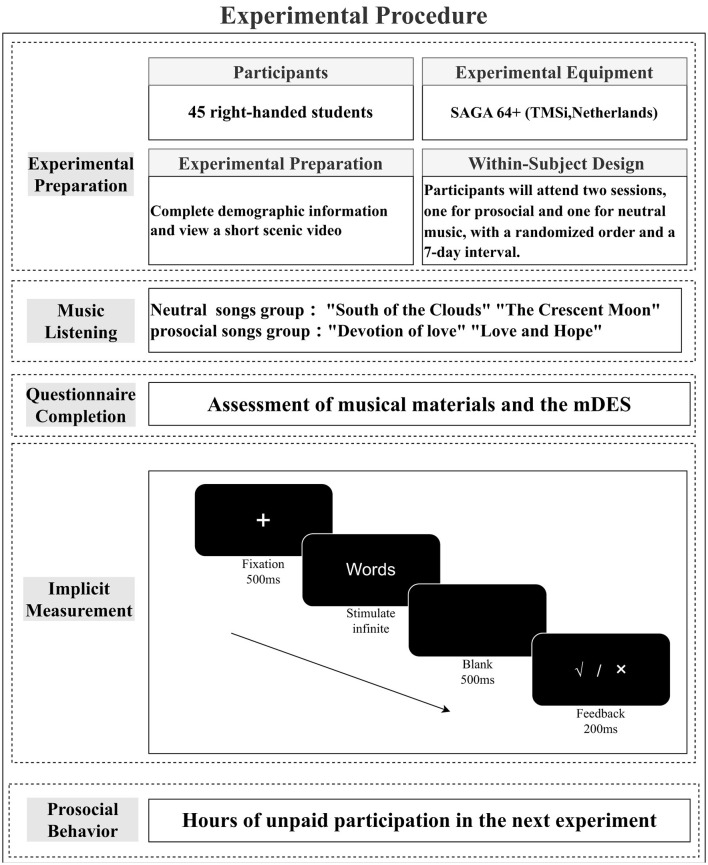
Experimental flow chart.

The experimental procedure (in Figure 2) will primarily consist of preparatory steps and listening to music materials. Participants will visit the laboratory twice, with a seven-day interval between the two sessions. In one session, they will listen to prosocial music, and in the other session, they will listen to neutral music, with the order of prosocial and neutral music being randomized. After each listening session, participants will complete relevant questionnaires and undergo the SC-IAT test to measure implicit prosocial cognition and assess prosocial behavior. Based on previous research (Yu et al., 2019), we will adopt the “voluntary participation in future experiments” paradigm as a measure of prosocial behavior. To prevent participants from making blind guesses about the “voluntary participation duration,” we explicitly informed them that after each session, they would be asked to complete a questionnaire to collect their willingness to participate in future experiments without obligation.

The SC-IAT will be conducted following exposure to both neutral and prosocial songs as stimulus materials. This approach will explore the influence of musical materials on implicit cognition and associated neural mechanisms. The experimental design adopts a mixed factorial design, comprising two factors: group (prosocial songs group, neutral songs group) and condition type (compatible condition, incompatible condition). After the music listening session and before the SC-IAT experiment, we will place the EEG cap on the participants and record the EEG signals.

The SC-IAT experiment, programmed using E-prime 2.0 following the method described by Karpinski and Steinman (2006), comprises two tasks: compatibility and incompatibility. Each task includes 20 practice trials and 80 experimental trials. In the compatibility task, participants use the “F” key for self or prosocial words and the “J” key for non-prosocial words. The roles of the keys are reversed in the incompatibility task. To avoid response bias, the distribution of word types is adjusted for a 1:1:2 ratio in the compatibility task and a 1:2:1 ratio in the incompatibility task. Participants receive feedback for 200 ms after each trial, with a green “√” for correct responses and a red “ × ” for incorrect ones. The order of tasks is counterbalanced across participants.

### 2.5 EEG recording and data processing

EEG data will be acquired using a 64-channel EEG cap based on the International 10–20 system arrangement, utilizing the SAGA 64 + system (TMSi, The Netherlands). An averaged reference will be employed during EEG recordings, ensuring that the contact resistance of electrode points remains below 5 KΩ, with a sampling frequency of 500 Hz.

Data analysis will be performed using Matlab and the freely available EEGlab toolbox (version 2021.0). All data will be re-referenced offline to averaged bilateral mastoids (M1, M2) and will be filtered using a 0.1–40 Hz bandwidth. Before segmenting the EEG data, EOG artifacts and electrical noise will be rejected manually from the raw data using independent component analysis. Preprocessed EEG data will be epoched 200 ms prior and 1,000 ms after the trigger events (i.e., the appearance of the target stimulus, such as prosocial or non-prosocial words), with baseline correction. Only correct trials will be included in the analysis. Based on previous studies (Carlson et al., 2016; Lou et al., 2021), we have selected electrode sites for three EEG components: N1, P200, and LPC. The N1 component (100–150 ms) will be recorded from electrodes frontal region (FC1, FCz, FC2) and central region (C1, Cz, C2); The N2 component(200–300 ms)will be recorded from the frontal region (Fz, F3, F4) and central region (Cz, C3, C4); The N4 component (350–450 ms) will be recorded from the frontal region (Fz, FCz), central region (Cz), and parietal region (CPz); The P200 component (148–200 ms) from the frontal region (F3, Fz, F4), fronto-central region (FC3, FCz, FC4), and central region (C3, Cz, C4).; and the LPC component (300–400 ms) from the parietal region (CP1, CPz, CP2, P1, Pz, P2) and occipital region (PO3, POz, PO4).

Paired-sample *t*-tests will have been used to analyze the D values of the SC-IAT after different music stimuli. A three-way (brain region × group × condition type) repeated-measures analysis of variance (RM ANOVA) will be used to process the mean amplitude of N1, N2, N4, P200, and LPC using SPSS 25.0 software. The degrees of freedom of the F ratio were corrected, according to the Greenhouse–Geisser method. Least square difference tests were performed as *post-hoc* analyses, if indicated.

## Author's note

Dan Yang is First Corresponding Author and Xiubo Ren is Second Corresponding Author.

## Data Availability

The original contributions presented in the study are included in the article/supplementary material, further inquiries can be directed to the corresponding author.
